# Dynamics of cerebral blood flow following sertraline treatment in adolescent depression

**DOI:** 10.3389/fpsyt.2025.1521565

**Published:** 2025-07-18

**Authors:** Wenyu Ren, Xiao Liu, Zhiwei Zhang, Xiaoqin Zhang, Wei Wu, Xingyu Wang, Zhihui Yu, Chuan Liu, Renqiang Yu

**Affiliations:** ^1^ Department of Radiology, The First Affiliated Hospital of Chongqing Medical University, Chongqing, China; ^2^ Department of Radiology, Bishan Hospital of Chongqing, Bishan Hospital of Chongqing Medical University, Chongqing, China

**Keywords:** depression, adolescent depression, cerebral blood flow, arterial spin labeling, medication, fMRI

## Abstract

**Background:**

Arterial spin labeling (ASL), a non-invasive magnetic resonance imaging (MRI) technique, has been employed to assess variations in cerebral blood flow (CBF) in adolescents diagnosed with depression. While prior studies have explored CBF abnormalities in depressed adolescents, the specific patterns of CBF changes following pharmacological interventions, particularly with selective serotonin reuptake inhibitors (SSRIs) such as sertraline, remain insufficiently characterized.

**Objective:**

To investigate the alterations in CBF induced by an 8-week sertraline treatment in adolescents with depression, and to assess whether baseline CBF can serve as a potential biomarker for predicting treatment response.

**Methods:**

A total of 40 adolescents diagnosed with depression and 31 age- and sex-matched healthy controls were enrolled in the study. Among the depressed cohort, 25 participants adhered to the treatment protocol and completed MRI scans. Resting-state functional MRI (rs-fMRI) scans were conducted for all participants, with a subsequent scan for the depression group after 8 weeks of sertraline therapy. Changes in CBF across various brain regions were examined using ASL data. The analysis and processing of ASL data were performed using Statistical Parametric Mapping 12 (SPM12) software and the MATLAB platform. Furthermore, Pearson correlation analysis was employed to examine associations between changes in regional CBF and clinical improvement, as measured by changes in the 17-item Hamilton Depression Rating Scale (HAMD-17) scores.

**Results:**

At baseline, adolescents with depression exhibited increased CBF in the posterior cuneus and decreased CBF in the middle temporal gyrus (MTG), right middle orbitofrontal gyrus, and the left middle frontal gyrus (MFG), compared to healthy controls. After 8 weeks of sertraline treatment, patients showed increased CBF in the right insula and decreased in the right MTG. Notably, baseline CBF in the left orbitofrontal gyrus was positively correlated with the magnitude of clinical improvement (i.e., reduction in HAMD-17 scores).

**Conclusion:**

The findings reveal significant differences in CBF between adolescents with depression and healthy controls. Moreover, alterations in CBF were observed in specific brain regions after an 8-week treatment regimen with sertraline, suggesting that these areas may be pivotal in the therapeutic effects of sertraline for treating adolescent depression. A decrease in HAMD-17 scores in the majority of treated patients underscores the efficacy of sertraline therapy. Notably, the change in HAMD scores from pre- to post-treatment was positively correlated with baseline CBF in the left MFG, indicating the potential of this region as a prognostic indicator.

## Highlights

- Utilization of ASL to non-invasively assess CBF changes in depressed adolescents.
- Significant CBF alterations in specific brain regions post-sertraline treatment.
- Identification of baseline CBF in the left MFG as a potential predictor of treatment response.

## 1 Introduction

Depression is a prevalent psychiatric disorder characterized by persistent low mood, anhedonia, diminished interest in daily activities, and impairments in cognitive and emotional functioning (1, 2). According to the Global Burden of Disease study, depression ranks as a leading contributor to disability and premature mortality worldwide (3). The incidence of depression among adolescents has gradually increased in recent years, with a prevalence rate of up to 20% throughout adolescence (4, 5). It has been shown that most adult depressions begin in adolescence and have a lifelong probability of developing, and major depression is a moderately hereditary condition (6–8). Since adolescence is an important time for physical, intellectual, and brain development (9), this disease may have a profound negative impact on the brain (10). However, the risk of these negative outcomes can be reduced over time with effective early treatment (11). Despite extensive research on depression, pathophysiological mechanisms, as well as the lack of objective diagnostic methods that are still based on subjective interpretation through clinical criteria, remain unclear (12). Recent research has revealed that traditional methods of assessing depression through scales have certain limitations, leading to advocacy for new evaluation standards. In this context, several studies (13, 14) demonstrated that self-assessment and clinical evaluations by physicians are complementary in quantifying the extent of depression. This finding stressed the importance of combining multiple assessment measures to more comprehensively and accurately diagnose and understand depression. Therefore, it is especially important to explore standards that can objectively assess adolescent depression and its treatment outcomes.

Selective serotonin reuptake inhibitors (SSRIs) are the first-line pharmacological treatment for adolescent depression, owing to their established efficacy, safety, and tolerability profiles (15–17). The primary mechanism by which SSRIs exert their therapeutic effects is through the inhibition of the presynaptic serotonin transporter (SERT), which prevents the reuptake of serotonin from the synaptic cleft. This action leads to an increased concentration of serotonin at serotonergic synapses, thereby amplifying serotonergic neurotransmission and contributing to mood stabilization and antidepressant effects (18, 19). Among SSRIs, sertraline has been widely prescribed in the adolescent population due to its favorable safety profile and relatively low incidence of side effects (20, 21). Emerging neuroimaging and neurobiological studies suggest that beyond modulating serotonin levels, SSRIs may also induce neuroplastic changes in the brain. Specifically, Radulescu et al. (22) argued that certain SSRIs facilitate the reorganization of neural networks by enhancing synaptic plasticity and promoting the restoration of dysfunctional circuits, particularly in emotion-regulation regions. These mechanisms are thought to involve not only serotonergic transmission but also modulation of glutamatergic signaling and synaptogenesis. In support of this neuroplasticity model, Johansen et al. (23) found that daily administration of escitalopram (an SSRI antidepressant) to healthy volunteers resulted in changes in synaptic plasticity in the brain after 3–5 weeks. This discovery clarifies, to some extent, why SSRI medications require a longer period to produce their antidepressant effects. Furthermore, Yang et al. (24) found that sertraline treatment in patients with depression significantly enhanced functional connectivity in key brain regions such as the prefrontal cortex and limbic system. Importantly, these changes were positively correlated with improvements in Hamilton Depression Rating Scale (HAMD) scores. This suggests that increased functional integration within emotion-related networks may serve as a neural correlate of symptom relief.

Functional neuroimaging has become a crucial tool in depression research. Although positron emission tomography (PET) and single-photon emission computed tomography (SPECT) have been initially used to measure cerebral blood flow (CBF) (25), their clinical and research applications—particularly in pediatric and adolescent populations—are limited due to several factors. These include relatively low spatial resolution, the necessity of using radioactive tracers which expose subjects to ionizing radiation, and the high operational and procedural costs (26–28). Arterial spin labeling (ASL) is an advanced, non-invasive magnetic resonance imaging (MRI) technique. It uses magnetically labeled arterial blood as an endogenous tracer to measure cerebral perfusion, providing quantitative measures of CBF (29, 30).

Although sertraline is one of the most widely studied SSRIs in the treatment of depression, the majority of existing research has focused on adult populations, with relatively fewer studies investigating its neurophysiological mechanisms in adolescents. In particular, limited attention has been given to how sertraline modulates CBF and functional connectivity in this age group. Yang et al. (31) reported that sertraline has a modulating effect on the hypothalamus-related functional connectivity in depression. In their subsequent study, they found that sertraline increased the functional connectivity strength of the network centered around the posterior cingulate cortex (24). Hsu et al. (32) demonstrated that sertraline can restore the normal functioning of default and emotion-related brain networks in patients with depression. Furthermore, the connectivity between the thalamus and prefrontal cortex (PFC) can predict the effectiveness of sertraline treatment, highlighting the potential of using brain connectivity as a predictive factor for treatment outcomes before the start of therapy. In a study on the treatment for depression with suicidal self-harm tendencies, Dai et al. (33) observed an upregulation of frontal lobe neuron activity and a downregulation of occipital lobe neuron activity. This suggests the effectiveness of sertraline in treating this condition. Further research on the effect of sertraline on CBF in adolescent depression patients is urgent. While evaluating treatment outcomes is a crucial objective in depression research, it remains unclear whether there is a specific correlation between baseline CBF measured through ASL and the brain regions that exhibit changes after sertraline treatment.

Herein, we advanced the hypothesis that the administration of sertraline — a selective serotonin reuptake inhibitor — induces modifications in CBF among adolescents with depression. These alterations in CBF are posited to correlate with observable clinical symptoms presented by the patients. The present study attempted to elucidate the underlying pathophysiological mechanisms of depression in the adolescent demographic, thereby contributing to a more nuanced understanding of the etiology and progression of this disorder. Furthermore, this study sought to delineate the therapeutic efficacy and relevance of sertraline treatment regimens in this specific patient cohort. By systematically examining the relationship between sertraline treatment and changes in CBF, as well as the corresponding clinical outcomes, this study endeavors to provide empirical evidence that will inform clinical practices and potentially guide the development of more targeted therapeutic interventions for adolescent depression. The conclusions derived from this research are anticipated to have significant implications for both the theoretical framework of depression pathogenesis and the practical application of pharmacological treatments in adolescent populations.

## 2 Materials and methods

### 2.1 Participants

Forty depression and 31 healthy control (HC) subjects aged 12–17 were recruited. All adolescents with depression were recruited from the Department of Psychiatry at the First Affiliated Hospital of Chongqing Medical University between April 2020 and March 2022. All cases were diagnosed with depression by two experienced clinical doctors using the Mini International Neuropsychiatric Interview for Children and Adolescents (MINI-KID), and the severity of symptoms was assessed using the 17-item Hamilton Depression Rating Scale (HAMD-17). The inclusion criteria of patients were as follows: (1) newly diagnosed depression patients who had not yet received any treatment, or those who had not received any medication or electroconvulsive therapy in the past month, (2) Han ethnicity, (3) aged 12–17 years, (4) right-handed, (5) those with at least a primary school education level, and (6) those with a HAMD-17 score > 17. The exclusion criteria of patients were as follows: (1) patients with concurrent other psychiatric disorders, (2) those with organic brain diseases or severe brain injuries, (3) those with serious physical illnesses, (4) a history of drug dependence or abuse, (5) those not actively cooperating with the researchers, and (6) those with contraindications for MRI. Patients were assessed at baseline and then re-evaluated after 8 weeks of treatment.

The HC group consisted of adolescents recruited from local schools between November 2020 and June 2022, matched in age, gender, and years of education with depression. The inclusion criteria included (1) Han ethnicity, (2) right-handed, (3) aged 12–17 years, (4) HAMD score < 7, and (5) exhibiting no serious mental or physical illnesses. The exclusion criteria were similar to those in the depression cohort.

This study was approved by The First Affiliated Hospital of Chongqing Medical University. Written informed consent was obtained from adolescents and their parents or legal guardians.

### 2.2 Intervention process

Sertraline was selected as the main antidepressant drug with an initial dose of 50 mg per day, and the maximum dose was 100–200 mg per day depending on the patient’s condition. The medication was administered for 8 weeks.

### 2.3 General data collection and evaluation indicators

All 40 adolescents with depression and 31 HCs were assessed via the HAMD-17 at baseline, and 25 adolescents with depression were re-assessed after 8 weeks of observation. All analyzed parameters were summarized as follows.

Sociodemographic data: This portion of the questionnaire enquired about the subject’s age, sex, and educational status.

Diagnostic assessment: Psychiatric diagnoses were confirmed using the Mini International Neuropsychiatric Interview for Children and Adolescents (MINI-KID), a structured diagnostic tool aligned with DSM and ICD criteria.

Depression severity: The HAMD-17 was employed to quantify depressive symptom severity. Assessments were conducted by two trained psychiatrists and scored independently by blinded raters to minimize bias. Symptom severity was classified using standard cutoffs (0tof no depression; 8epre mild; 18ld;s moderate; >25: severe).

Therapeutic response: Significant therapeutic effectiveness was described as ≥75% reduction in the HAMD score or a sum score of ≤7 points following intervention. Effective therapy was defined as a 50-75% reduction in the HAMD score, improvement was defined as 25-50% reduction in the HAMD score, and invalid was defined as <25% reduction in the HAMD score (34).

### 2.4 MRI image data acquisition

All neuroimaging data were meticulously acquired using the advanced Signa 3.0 Tesla MRI system (GE Medical Systems, Waukesha, WI, United States). The initial scanning session encompassed the acquisition of T1-weighted images, ASL data, and T2-Fluid-Attenuated Inversion Recovery (T2-FLAIR) sequences. Subjects presenting with abnormalities evident in T2-FLAIR images were deemed unsuitable for the objectives of the study and were subsequently excluded. Following exclusion, these individuals were referred to the Department of Neurology for comprehensive evaluation and, if necessary, intervention.

To ensure the integrity of the imaging data, participants were instructed to adopt a supine position within the MRI scanner and adhere to a protocol designed to minimize physiological noise and motion artifacts. This protocol required participants to remain still, with their eyes closed, and to avoid engaging in any specific cognitive or mental activities, thereby maintaining a relaxed yet awake state. The auditory protection was provided through the use of earplugs, and head motion was minimized with the strategic placement of a sponge, further ensuring the quality of the acquired data. Imaging was conducted using a standard eight-channel head coil. The MRI examination was repeated after 8 weeks of treatment.

The MRI protocol was meticulously designed to exclude structural abnormalities and intracranial lesions, with particular attention paid to the acquisition of three-dimensional (3D) T1-Weighted Imaging (T1WI), T2-Weighted Imaging (T2WI), and T2 FLAIR sequences. The following parameters for the 3D T1-weighted anatomical scans were carefully selected to optimize image quality and resolution: a repetition time (TR) of 8.4 milliseconds, a flip angle of 12°, an echo time (TE) of 3.3 milliseconds, a field of view (FOV) of 24 × 24 cm, and a matrix size of 256 × 256, resulting in 156 axial slices with a slice thickness of 1 mm. Furthermore, resting-state perfusion imaging was conducted using 3D ASL techniques, with the following parameters: a number of excitations (NEX) of 3, a TR of 4639 milliseconds, a TE of 9.8 milliseconds, a FOV of 240 × 240 mm, a slice thickness of 4.0 mm, a post-label delay time of 1525 milliseconds, and a 3D spiral k-space filling technique with 512 points and 8 arms, covering 40 slices. After the examination, whether the patient remained awake throughout the entire procedure was confirmed.

### 2.5 Image processing

The dcm2nii software (https://neuroelf.net/wiki/doku.php?id=dcm2nii) was used to convert the CBF digital imaging and communications in medicine (DICOM) images to neuroimaging informatics technology initiative (NIFTI) formats before analysis using Statistical Parametric Mapping 12 (SPM12) in MATLAB 2013b. The preparatory stages encompassed motion adjustment, aligning functional to structural images, conforming images to a standard space, and applying a smoothing filter. For ASL image motion adjustment, participants displaying head movements greater than 2 mm in shift or 2° in pivot were not included. ASL pictures were aligned with the T1 structural scans for standardization to the Montreal Neurological Institute (MNI) coordinates. The aligned scans were then mapped to the default MNI template in SPM8. The images were then standardized using dpabi4.3 (http://rfmri.org/dpabi). ASL pictures underwent a smoothing process using a Gaussian filter with a 6-mm full-width at half-maximum (FWHM) to enhance the signal clarity.

### 2.6 Statistical analysis

SPSS software (version 26.0, IBM, Chicago, New York, USA) was deployed for data analysis to assess the clinical and demographic characteristics distinguishing depressed individuals from HCs. To minimize the influence of potential confounding factors on CBF measures, we incorporated age, gender, and educational background as covariates in all group-level statistical models. These variables were selected based on their known associations with neurodevelopment and brain function. In particular, educational background has been linked to differences in cognitive performance and cortical organization, both of which may influence resting-state perfusion metrics, independent of disease status. Specifically, a whole-brain two-sample t-test was performed using SPM12, with these factors integrated as covariates in a general linear model. Furthermore, paired t-tests comparing pre- and post-treatment CBF within the depression group were adjusted for these covariates to maintain consistency. The preliminary criterion for analysis was set at a voxel-level significance threshold of p < 0.05, followed by an adjustment for multiple comparisons at the cluster level, applying a Family Wise Error (FWE) correction with a threshold of p < 0.05.

The inquiry was expanded to include an assessment of variations in the severity of depression using a paired t-test. Subsequently, regions in the brain affected by gender and group interactions for CBF perfusion were determined using xjView software (https://www.alivelearn.net/xjview). The brain area exhibiting interactive effects was designated as the region of interest (ROI), from which the CBF value was extracted utilizing DPABI software. Significant changes in the clinical symptoms of adolescent depression, as indicated by HAMD scores, were then correlated with CBF metrics for a focused analysis of specific cerebral regions through Pearson correlation analysis (PCA). PCA was also utilized to investigate the potential significant relationship between CBF metrics and the extent of change in clinical symptoms (ΔHAMD = pre-treatment HAMD – post-treatment HAMD), with p < 0.05 designated as the threshold for significance.

## 3 Results

### 3.1 Patient prognosis

The demographics of the participants are detailed in **Table 1**. No significant differences were observed between adolescents with depression and HCs in terms of gender, age, and education level (p > 0.05). Both MRI data and responses to questionnaires were collected from 25 individuals with depression who were successfully followed up before and after 8-week treatment with sertraline. It was found that 20% (5/25) of the subjects experienced significant therapeutic benefits, 24% (6/25) responded positively to the treatment, 32% (8/25) showed signs of improvement, and 24% (6/25) did not respond to the treatment. Meanwhile, HAMD scores reduced dramatically, with a strong statistical significance (p < 0.001) (**Table 2**).

**Table 1:** Demographics and clinical profiles of adolescent depression and healthy controls (HCs).

Item	Depression group (n=40)	HCs group (n=31)	*t*/*X* ^2^	*P*
Gender (F/M)	11/29	9/22	0.02^a^	0.887
Age(years)	15.18 ± 1.430	15.26 ± 1.897	0.21^b^	0.834
Education(years)	9.55 ± 1.535	9.94 ± 2.308	0.843^b^	0.402
HAMD	24.13 ± 3.963	1.23 ± 1.802	-29.837^b^	<0.001

The values are illustrated as mean ± SD.

a chi-square test.

b two sample t-test.

**Table 2:** Comparisons of clinical symptom severity between pre- and post-therapeutic intervention.

Variable	pre-ECT (n=25)	post-ECT (n=25)	*t*	p valve
HAMD	23.60 ± 3.80	13.92 ± 6.41	6.50	< 0.001

The values are illustrated as mean ± SD. P value is obtained by paired t-test.

### 3.2 Differences in baseline CBF between adolescents with depression and HCs

**Figure 1:**
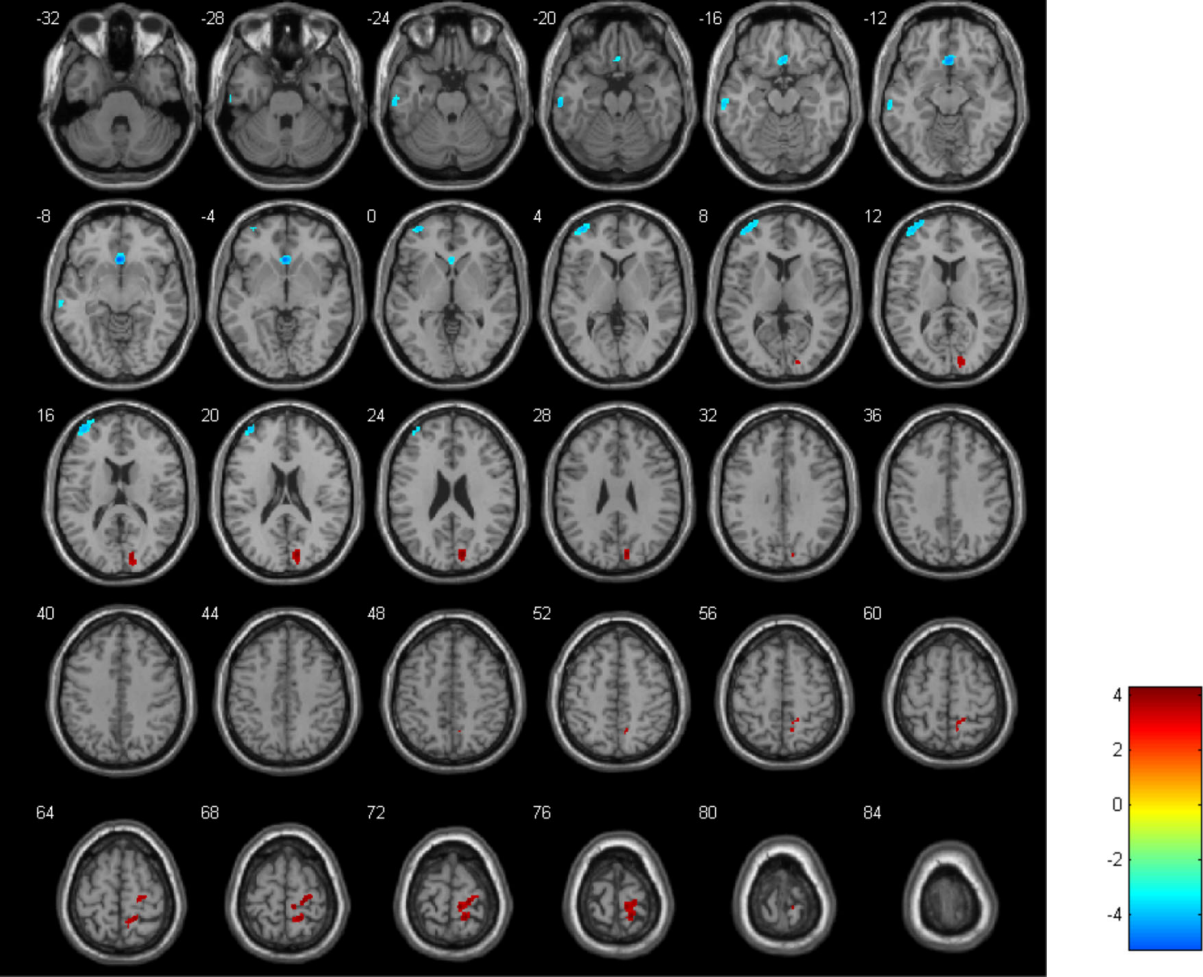
Visualization of variations in cerebral blood flow (CBF) differentiating adolescents with depression from HCs. Blue zones represent diminished CBF and the red zones indicate increased CBF.

Compared with HCs, depressed adolescents showed significant decreases in CBF in the left middle temporal gyrus (MTG) (with a cluster size of 152 and a peak t-value of -3.99), the right middle orbitofrontal gyrus (mOFG) (with a cluster size of 250 and a peak t-value of -5.22), and the left middle frontal gyrus (MFG) (with a cluster size of 383 and a peak t-value of -4.25). Conversely, CBF was increased in the right cuneus (with a cluster size of 230 and a peak t-value of 4.25) and the right postcentral gyrus (PoCG) (with a cluster size of 480 and a peak t-value of 4.21) (**Figure 1**). The criterion for analysis was set at a cluster-level significance threshold of p < 0.001.

Notably, significant correlations were observed at baseline between HAMD-17 scores and CBF in specific brain regions when considering the full sample of 25 depressed adolescents. However, these associations did not persist after the 8-week sertraline treatment, suggesting a potential decoupling between regional perfusion and symptom severity following pharmacological intervention, irrespective of individual treatment response status.

### 3.3 Differences in CBF between the pre- and post-treatment patients

**Figure 2:**
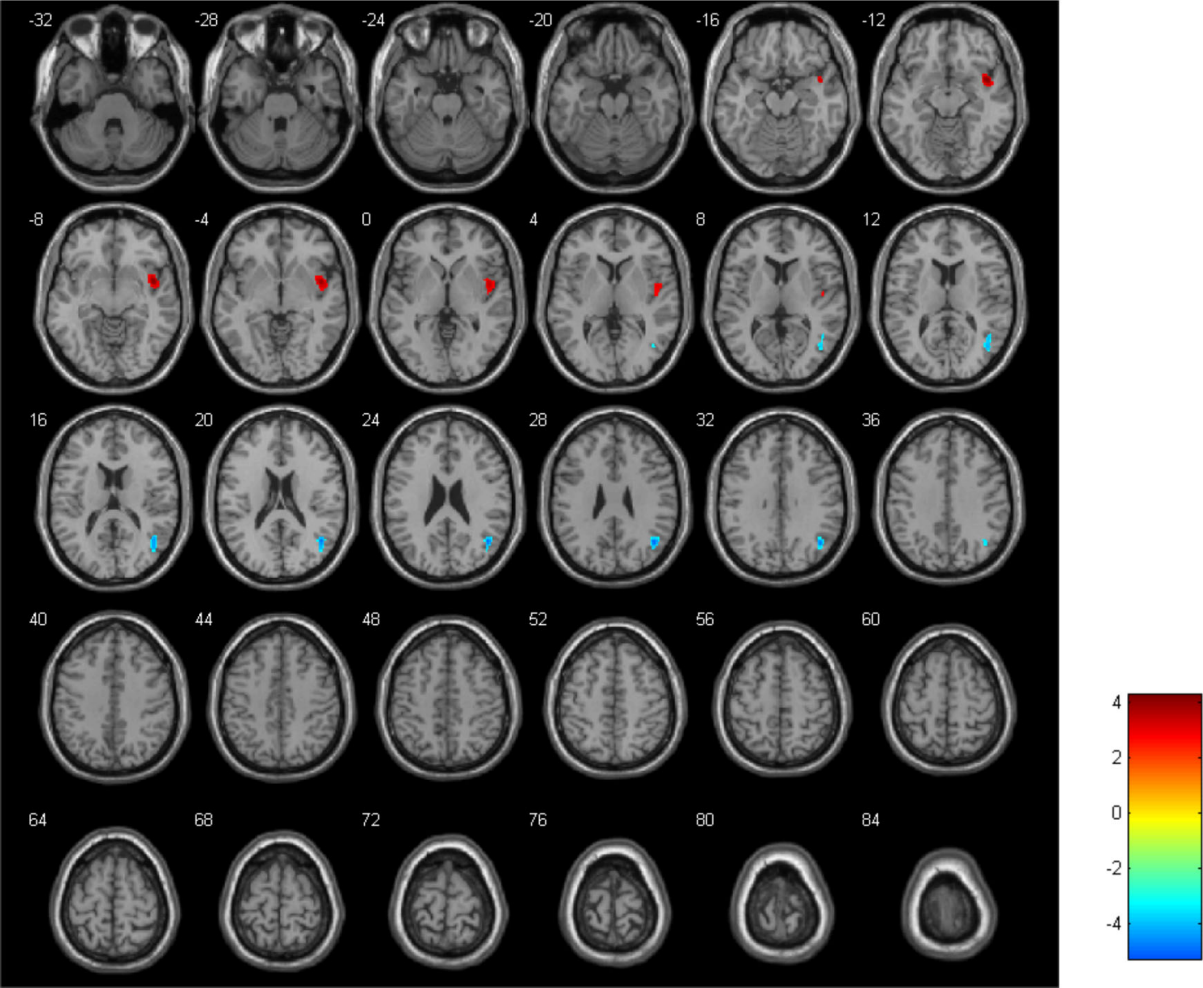
The CBF alterations in adolescents with depression before versus after undergoing therapeutic intervention, with blue indicating a decrease and red an increase in CBF.

The results revealed a reduction in CBF in the right MTG (with a cluster size of 357 and a peak t-value of -5.87) but an increase in CBF in the right insula (INS) (with a cluster size of 347 and a peak t-value of 5.56) after 8 weeks of sertraline treatment (**Figure 2**). The criterion for analysis was set at a cluster-level significance threshold of p < 0.001.

### 3.4 Correlation between the CBF values and ΔHAMD scores

**Figure 3:**
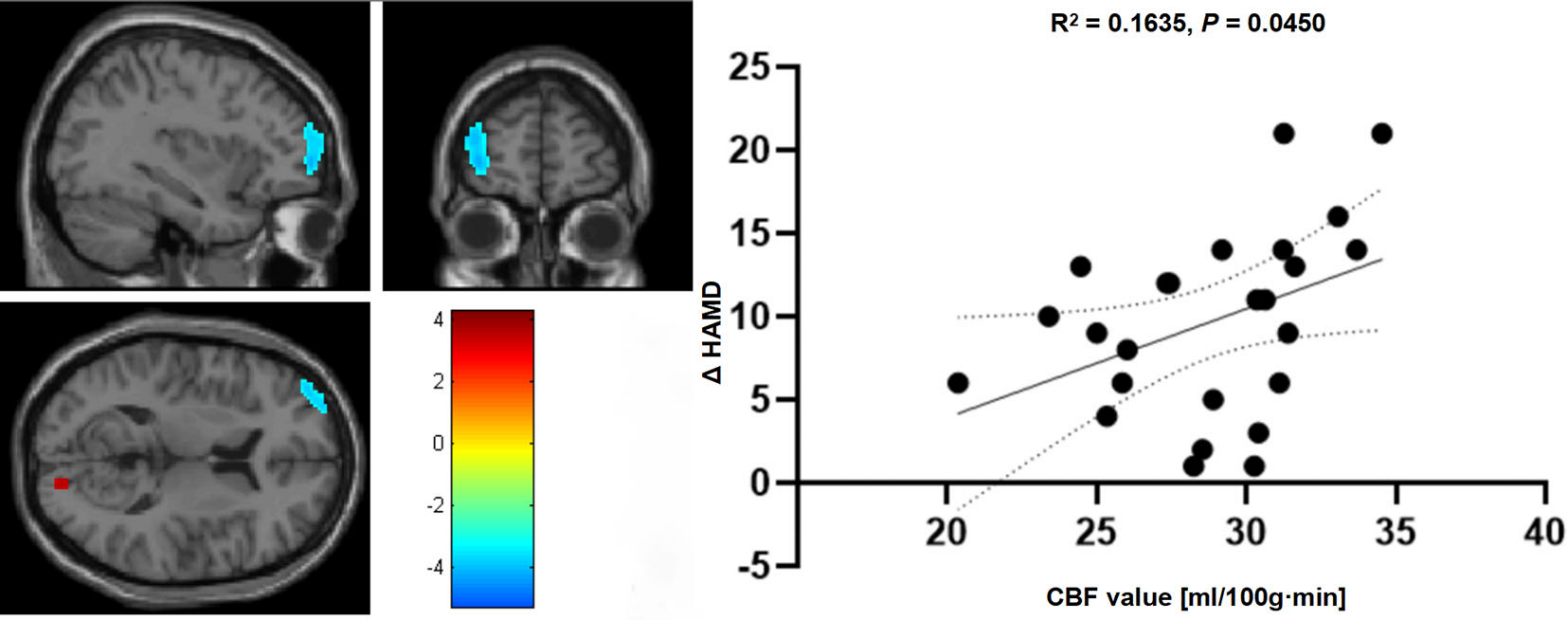
The relationship between CBF measurements within the left MFG in comparison to changes in ΔHAMD scores before and after therapeutic intervention in adolescents with depression.

PCA revealed a positive correlation between CBF data in the left MFG and ΔHAMD scores, when comparing pre-treatment data in adolescents (R² = 0.164, p = 0.045) (**Figure 3**). This correlation suggests that alterations in CBF in the left MFG are significantly linked to changes in depression severity following treatment. However, no significant correlations were observed between ΔHAMD scores and CBF data in other brain regions.

## 4 Discussion

To the best of our knowledge, few studies, including ours, have delved into the examination of CBF in depressed adolescents undergoing treatment with sertraline. The current study utilized resting-state functional magnetic resonance imaging (rs-fMRI) to discern variances in CBF in adolescents with depression compared with HCs. Initially, subjects with depression exhibited reduced CBF in areas such as the MTG, right mOFG, and left MFG and heightened CBF in the right cuneus and right PoCG. The study highlighted that 8-week treatment with sertraline significantly relieved symptoms in 44% (11/25) of the participants. Post-treatment observations revealed a decrease in CBF in the right MTG, which was elevated in the right INS. Additionally, CBF in the left MFG was positively correlated with ΔHAMD scores.

According to previous studies, the characteristics of CBF in patients with depression include decreased regional perfusion in areas such as the right parahippocampal gyrus, thalamus, fusiform gyrus, middle temporal gyrus (MTG), and bilateral insulae (35), as well as increased CBF in frontal, temporal, and parietal lobes (36). These findings differ from our results, and such inconsistencies may stem from differences in study populations (e.g., adult vs. adolescent subjects), variations in disease duration, and differences in imaging or analytic methodologies. Additionally, it is possible that treatment-related factors or the dynamic nature of depressive pathology may contribute to variability in regional cerebral perfusion, although this remains speculative and requires further investigation in longitudinal and developmental contexts.

A study found that drug-induced CBF reduction occurred in areas of the limbic system, including the amygdala, insula, and orbitofrontal cortex (OFC) (37). This may be due to differences in the characteristics of the study population and the choice of medication. It can also be inferred that effective target brain regions for drug action in adolescent depression patients may differ from those in other groups. Yang et al. (31)study showed that the connectivity between the hypothalamus and various brain regions was enhanced after 8 weeks of sertraline antidepressant treatment, including areas such as the insula and caudate nucleus. A specific functional connectivity pattern in the right insula can predict the response to sertraline (38). The insula is a network associated with interoception, emotional self-awareness, and decision-making (39). Sertraline plays a significant role in improving depressive moods and anxiety symptoms. In this study, we observed increased CBF in the insular region following treatment, alongside clinical improvement. While the insula is a key node in affective and salience networks, the precise neurophysiological mechanisms underlying this regional perfusion change remain unclear. Future research could investigate whether changes in CBF are associated with, or potentially mediate, alterations in large-scale brain network connectivity in response to antidepressant treatment.

Although the mechanism of action of sertraline in treating depression is different from that of transcranial magnetic stimulation (TMS), psychotherapy, and other methods, it also helps to normalize abnormal local CBF in depressed patients; the normalization of abnormal CBF patterns represents the effectiveness of antidepressant treatment (40). Areas of normalized CBF after treatment include the left MTG and the post-central gyrus. The MTG is a brain region involved in social cognitive processing and emotional information processing. Suicidal ideation is one of the characteristic symptoms of depression, and the MTG is associated with suicidal ideation in depression through neuroimaging studies (41, 42). A study of adolescent depression identified increased resting-state functional connectivity between the amygdala and bilateral temporal lobes in the patient group and decreased functional connectivity between the two in the HC group (43). A follow-up study also found that CBF in the inferior temporal gyrus, MFG, inferior frontal gyrus, and knee of the anterior cingulate gyrus significantly returned to normal levels after 6 weeks of citalopram treatment in depressed patients (44). The temporal lobe is involved in regulating mood in the brain. Abnormalities in temporal lobe function can lead to abnormal mood regulation, providing evidence for the involvement of the temporal lobe in the development of depression.

However, sertraline does not normalize all brain regions with abnormal CBF. In a study of CBF alterations in patients with late-life depression, CBF increased in the left dorsolateral PFC to precentral and right parieto-occipital regions after antidepressant treatment. However, CBF in regions such as the anterior ventral/dorsal medial PFC did not improve significantly, reflecting underlying and persistent cerebral dysfunction associated with depression (45).

The OFG is an important region of the human brain involved in cognition, emotion regulation, motivation, and decision-making (46). Posterior medial and lateral parts of the OFG are involved in emotion regulation. Dysfunction in these regions increases the likelihood of major depression. In addition, certain physiological activities in these two regions are positively correlated with the severity of depression (47). Chiappelli and co-workers (Chiappelli et al., 2023) showed that trait depression was associated with lower CBF in the cingulate gyrus and frontal white matter.

The MFG is critically involved in motivation and reward processing. Patients with depression often exhibit reduced CBF in the frontal cortex, which is associated with impaired executive and affective function. Interventions such as repetitive transcranial magnetic stimulation (rTMS) have been shown to increase oxyhemoglobin concentrations in the frontal cortex, suggesting enhanced perfusion and neuronal activation (48). Structural studies also indicate that greater MFG volume is associated with lower depression severity (49). In the current study, we observed a significant positive correlation between baseline CBF in the left MFG and reduction in HAMD scores following sertraline treatment, implying a potential link between regional perfusion and symptom improvement.

Although this correlation is intriguing, it does not establish a predictive relationship. Additional analyses using predictive modeling techniques would be required to determine whether baseline MFG perfusion can reliably forecast treatment outcomes (50). Nonetheless, prior studies have suggested that neuroimaging biomarkers, including perfusion patterns (51), may inform personalized antidepressant strategies (52, 53). Future studies should investigate whether baseline CBF in regions such as the MFG can be incorporated into clinical decision-making frameworks to optimize treatment selection and improve response rates in adolescent depression.

Our findings underscore the significant potential of CBF, particularly in the left MFG, as a biomarker for both the pathophysiology of depression and treatment efficacy. While these results do not yet establish CBF as a definitive biomarker for clinical use, they suggest that perfusion imaging may contribute valuable insights into individual differences in therapeutic outcomes. In the future, integrating CBF measurements into clinical workflows could support the development of more precise, personalized treatment strategies—allowing clinicians to better monitor neurobiological changes associated with symptom improvement and to tailor interventions in a timely and adaptive manner. However, further longitudinal and predictive modeling studies are necessary to validate these applications in real-world settings.

## 5 Limitations

This study has several limitations that should be acknowledged. Firstly, the relatively small sample size in this study, with only 25 patients completing follow-up, represents a significant limitation that may restrict the generalizability of our findings.

In addition, this trial adopted an open-label approach for practical reasons. Thus, the absence of blinding might have introduced potential for bias. Lastly, while our results suggest overall improvement in depressive symptoms following sertraline treatment, we acknowledge considerable variability in individual response profiles. Due to the limited sample size within each response subgroup, we did not perform stratified imaging analyses, which may have obscured neurobiological differences associated with treatment efficacy. Future studies with larger samples are needed to investigate differential neural correlates of treatment response and to better understand the heterogeneity in antidepressant outcomes among adolescents.

## 6 Conclusion

Our study found significant changes in CBF in adolescents with depression. Furthermore, administration of sertraline, a common antidepressant medication, led to a noticeable decrease in HAMD-17 scores in a group of 25 adolescents. These findings suggest that sertraline may be effective in treating depressive symptoms in this age group. The impact of sertraline on the neural architecture of these patients was further elucidated through rs-fMRI, which yielded strong evidence of neuroimaging changes post-treatment. Specifically, post-sertraline intervention, an upsurge in CBF within the insular cortex coupled with a reduction in the middle temporal gyrus, was observed, indicating significant neurophysiological adjustments. Interestingly, the improvement in HAMD-17 scores after treatment was linked to higher blood flow in the left MFG before treatment. This suggests that baseline blood flow in this brain region might be a predictor of how well a patient responds to treatment.

## Data Availability

The raw data supporting the conclusions of this article will be made available by the authors, without undue reservation.
